# Citizen science approach for springshed management: A comprehensive community-driven mapping and dataset of spring sources in Kavre, Nepal

**DOI:** 10.1016/j.dib.2025.111466

**Published:** 2025-03-15

**Authors:** Srijan Thapa, Anju Pandit, Sanjeev Bhuchar, Madhav Dhakal

**Affiliations:** International Centre for Integrated Mountain Development (ICIMOD), Kathmandu, Nepal

**Keywords:** Springs, Citizen science, Community resource persons (CRPs), Inventorization, Springshed management

## Abstract

In the Himalayan region, a reliable and comprehensive spring source dataset is crucial for sustainable water source management, protection of the environment, climate change adaptation efforts, and formation of evidence-based informed policy decisions, where springs play a vital role in local water security. Community Resource Persons (CRPs)- these are local community members, who were trained in springshed management as well as in field data collection and collected the data using a mobile-based ArcGIS Survey123 app. The dataset includes the spring's georeferenced location, water use patterns, source conditions, discharge, basic socio-economic characteristics of dependent communities, and adaptation measures implemented by communities to address water scarcity. By integrating local knowledge and citizen science approaches with state-of-the-art digital technology, the dataset offers valuable insights for sustainable water resource management, highlighting the biophysical, cultural, social, and governance aspects of springs in the drought-prone region. This data can support socio-ecological research inform policymaking targeting water security and efforts aimed at the revival of springs. As the dataset is made publicly available through ICIMOD's Regional Database System (RDS) (https://rds.icimod.org/), it will serve as a foundation for the identification of critical springs for long-term monitoring and revival and assessing the impact on water security after the revival of dried springs, which will be important for researchers, policymakers, and conservation practitioners alike.

Specifications Table

**Table:** 

Subject	Water Science and Technology, Hydrology and Water Quality, Nature and Landscape Conservation, Anthropology, Sociology, Ecology, Sustainable Springshed Management, Citizen Science
Specific subject area	Comprehensive spring source mapping and documentation through a citizen science approach.
Type of data	Status of Springs Source Table
Data collection	The Data were collected using the ArcGIS Survey123 app for mobile-based mapping. Community Resource Persons (CRPs) mapped spring sources including spring location information, water discharge, source conditions, discharge trends, the status of springs, and measures adopted by the communities after drying of their water sources. The total mapping of 5,689 water sources over 743 square kilometres provides a detailed inventory of the region's water resources, including (5,168 springs) and (521 ponds). The data also contain basic socio-economic details of spring water-dependent communities.
Data source location	Regional Database System (RDS)Institution: International Centre for Integrated Mountain Development • City/ Town/Region: Khumaltar, Lalitpur, Kathmandu • Country: Nepal • Web: https://rds.icimod.org/
Data accessibility	Repository name: Status of Springs in Seven Municipalities of Kavre, Nepal Data identification number: https://doi.org/10.26066/RDS.1974131 Direct URL to data: https://rds.icimod.org/Home/DataDetail?metadataId=1974131 Instructions for accessing these data: To access the "Status of Springs in Seven Municipalities of Kavre" dataset, visit the Regional Database System (RDS) at https://rds.icimod.org. Users are required to register on the RDS website by providing minimal basic information. The dataset is available in .xlsx format and is accompanied by a metadata file that includes essential details about the fields.
Related research article	No

## 1 Value of the Data

• The dataset provides detailed information on spring sources, including GPS locations, one-time discharge measurements, and basic socio-economic, biophysical, cultural, social, and governance information. This comprehensive data set can support various research applications like groundwater modelling, climate change impact studies, and watershed management, by offering valuable insights into hydrological dynamics, resource availability, and community interactions with water sources.
• Spring source mapping is the first step in revival efforts, so it serves as a basis for identifying critical springs for long-term monitoring and revival. By dataset integration with other datasets like GIS, land use land cover change and climate data, can be applied to support long term trend assessment along with adaptive water resource management.
• The dataset integrates scientific research with local knowledge, which provides a framework for sustainable water management and the formulation of informed policy decisions, as some municipalities have included springshed management in their annual plans and policies. It also offers excellent potential for demonstrating value for money spent on efficient use of resources, scalability and adaptability to different geographical and socio-economic settings.
• This dataset offers a localized perspective/knowledge as it is collected by a community resource person that other researchers can replicate to enhance community participation in sustainable water management while also addressing gender-specific implications, social equity, community empowerment, indigenous knowledge perspective, and local capacity building approach, makes it a replicable model for other researchers.

## 2 Background

Springs in the Hindu Kush Himalaya (HKH) region play a vital role in ensuring rural water security, being major sources of water for household activities, drinking, irrigation, livestock, agriculture, and many other purposes [[1], [2], [3]]. Around 80 % of the 13 million people in Nepal's mountains and hills rely on spring water as the primary source of water [4].

Various research studies throughout the HKH region have documented a declining spring discharge or the complete drying up of springs, resulting in widespread water shortages and increased vulnerability for local communities. Natural causes such as climate changes, erratic precipitation patterns, drought, and natural disasters, along with anthropogenic activities such as infrastructure development and catchment degradation, have disrupted the hydrology in Nepal's Middle Hills, which escalated the drying of springs, particularly during the dry season [[4], [5], [6]]. This leads to reduced availability for drinking, agriculture, and household use, increased domestic drudgery with more time spent fetching water from distant sources, and negative impacts on public and ecological health [1]. In any region, a complete understanding of the status of water sources is essential for effective and sustainable water resource management [7]. Gaps remain in mapping and understanding the status of essential water sources in Nepal [8,9], despite advancements in spring research. The accurate documentation of the status of springs is important to address research gaps, guiding evidence-based policies as well as management strategies to ensure sustainable use and conservation.

Our research focuses on training community resource persons (CRPs), both women and men, to map springs and ponds addressing the above-mentioned research gap. This approach efficiently documents spring sources while providing a comprehensive understanding of their biophysical (hydrological, and ecological), cultural, social, and governance characteristics cost-effectively and in a shorter timeframe than conventional methods.

## 3 Data Description

The survey form is divided into a total of 7 sections for active spring/pond sources. For dried spring/pond sources, only 4 sections need to be filled.

### Active springs/ponds

#### Section 1: Enumerator information

*This section collects basic details about the enumerator, including their name, gender, and mobile number.*

#### Section 2: Location of water source

*As shown in* Table 1 *, which records the location details of the spring source, including the district, municipality, ward number, village name, and tole.*

**Table 1:** Location of water source.

*Question*	*Input Type*	*Option*
*District*	*Text field*	*Kavrepalanchowk*
*Municipality*	*Radio button*	*Bethanchok, Dhulikhel, Namobuddha, Panauti, Panchkhal, Roshi, Temal*
*Ward number*	*Numerical text field*	*-*
*Village name*	*Text field*	*-*
*Tole* *(****Tole****is a smaller neighbourhood within a****Ward****, representing a cluster of households or a local community.)*	*Text field*	*-*

#### Section 3: Information about the water source

This section records the details about the spring or pond, such as GPS coordinates, source name, source type, water source ownership, surrounding environment (land cover), plant species near the source, source condition (active or dried), and water source seasonality as illustrated in Table 2.

**Table 2:** Information about the water source.

*Questions*	*Input Type*	*Options*
*GPS locations of source*		*Automatically captured by the app*
*Source name*	*Text field*	*-*
*Source type*	*Radio button*	*Spring, pond*
*Source ownership*	*Radio button*	*Private, public, community*
*Surrounding Environment (Land Cover)*	*Radio button*	*Dense forest, open forest, agricultural land, grassland, fallow land, settlement, other*
*Plant species around water source (3 key species)*	*Text field*	*-*
*Source condition*	*Radio button*	*Active, dried*
*Source seasonality*	*Radio button*	*Perennial, seasonal*

#### Section 4: Questions about the spring/pond

*This section gathers detailed information about the condition of the spring or pond, including:*

• ***Spring questions:*** *Condition of the source, nature of discharge, water emergence point, volume measurements, perception of discharge variation over time, and distance from households as shown in* Table 3 *.*

**Table 3:** Spring related question.

*Questions*	*Input type*	*Options*
*Condition of source*	*Radion button*	*Cemented tank, open kuwa, closed kuwa, tap/natural flow, stone spout* *(Kuwa refers to traditional structures for collecting spring water, typically small open or covered pits, commonly found near natural springs in the Himalayan region.)*
*Nature of discharge*	*Radion button*	*Seepage, flowing*
*Spring/Water emergence point*	*Radion button*	*Rock, soil/sediment, interface between rock and sediment*
*Volume of bucket/bottle (1, 2, 5, 10 or 20 liter)*	*Numerical text field*	*-*
*Time to fill (seconds)*	*Numerical text field*	*-*
*Approx. distance from the nearest dependent household (meter)*	*Numerical text field*	*-*
*Two-way water fetching time (minutes)*	*Numerical text filed*	*-*
*How much time did it take to fill the jar of 20 liter 10 years ago? (minutes)*	*Numerical text field*	*-*
*Time to fill 20-liter jar (active) (minutes)*	*Numerical text field*	*-*
*Perception on trend of discharge*	*Radio button*	*Increasing, decreasing, same*
• ***Pond questions:*** *Current area and depth of the pond, as well as how these have changed over the past 10 years as shown in* Table 4 *.*

**Table 4:** Pond related questions.

*Question*	*Input Type*	*Options*
*Pond area (square meter)*	*Numerical text field*	*-*
*Pond depth (meter)*	*Numerical text field*	*-*
*Pond area 10 years earlier (square meter)*	*Numerical text field*	*-*
*Pond depth 10 years earlier (meter)*	*Numerical text field*	*-*

***Spring related question***

***Pond related questions***

#### Section 5 and 6: Social context

*These sections gather detailed information on social aspects related to the spring or pond as shown in* Table 5 *.*

**Table 5:** Social context questions.

*Question*	*Input Type*	*Options*
*Perception on source water quality*	*Radio button*	*Excellent, good, poor*
*Perception on source water quality change*	*Radio button*	*Improved, degraded, no change*
*Is this source in use?*	*Radio button*	*Yes, no*
*Purpose of use (multiple choice)*	*Check box*	*Drinking, household, recreation, agriculture, livestock, religious purpose*
*Number of households dependent on this source*	*Numerical text field*	*-*
*Number of households dependent on this source only?*	*Numerical text field*	*-*
*Ethnicity of users*	*Check box*	*BCTS, Newar, Janajati, Dalit, Mixed*
*Ethnicity of users dependent on this source only?*	*Check box*	*BCTS, Newar, Janajati, Dalit, Mixed*
Any impact due to 2015 earthquake?	*Radio button*	*decreased after earthquake, dried after earthquake, increased flow after earthquake, no change*
*Is this source tapped?*	*Radio Button*	*Yes, no*
*Supplies water to this municipality or to another municipality?*	*Radio Button*	*Same, different, both*
*Supplies water to this ward or to other wards?*	*Radio button*	*Same, different, both*
*Is water source significant for cultural/religious purpose?*	*Radio button*	*Yes, no*
*List the events associated with the source.*	*Text field*	*-*
*Is this source accessible to everyone in the community? (in terms of use access)*	*Radio button*	*Yes, no*
*If not, who are restricted?*	*Text field*	*-*
*Are there water user groups for source management?*	*Radio button*	*Yes, no*
*User group type*	*Radio button*	*Formal, informal*
*Who is chairperson?*	*Radio button*	*Male, female*
*Women in other leadership positions*	*Check box*	*Vice chair, treasurer, secretary, no position*
*Any spring revival/pond protection measures taken?*	*Radio button*	*Yes, no*
*What measures were taken?*	*Check box*	*Recharge pond construction, trench-pit, plantation, source protection*
*Remarks (source specific comments)*	*Text field*	-

#### Section 7: Submission page with photograph

A photograph of the spring source is mandatory, while up to two additional photos are optional. The survey can be submitted immediately if you are online or saved locally in the outbox on your device to be sent later when you have internet access.

### Dried springs/ponds

#### Section 1: Enumerator information

*This section collects basic details about the enumerator, including their name, gender, and mobile number.*

#### Section 2: Location of water source

*This section is the same as for active springs/ponds, as described in*Table 1: Location of water source*.*

#### Section 3: Information about the water source

This section includes all the questions from Section 3 for active springs/ponds shown in Table 2 along with additional questions related to dried sources that gather data on the duration and causes of drying, any revival activities, and actions taken after the source dried as shown in Table 6.

**Table 6:** Additional questions related to dried sources.

Question	Input type	Response
Dried since which year in Bikram Sambat (BS)? (Note: BS calendar is the official calendar of Nepal and runs approximately 56 years and 8.5 months ahead of the AD).	Numerical text field	-
Reason for drying?	Radio button	Drought, infrastructure development, earthquake, source disruption, natural disaster, deforestation, source neglected
After this source dried up, was there any activity to revive this source?	Radio button	Yes, no
List the activity done for source revival.	Check box	Recharge pond construction, trench-pit, plantation, source protection
What was done after source dried up?	Check box	New source identification, well, boring, water lifting, rainwater harvesting, tanker dependence, migration

#### Section 4: Submission page with photograph

*This section is the same as Section 7 of active springs/ponds.*

## 4 Experimental Design, Materials and Methods

### 4.1 Study area identification

The area of investigation was driven by local community demands together with implementing agencies based on available information. Kavrepalanchowk district is recognized as a drought-prone region [10]. Despite significant research on spring water resources across Nepal, there are notable documentation gaps in central regions like Kavre [9]. To address this gap, seven municipalities of Kavre district was selected for the inventorization of spring sources through focused research with engaging local communities.

### 4.2 Stakeholder consultation

The first step involved consulting with stakeholders, specifically local government elected representatives, including the chairs, vice chairs of municipalities and rural municipalities, ward chairs, and women representatives from each ward. These individuals were briefed on the importance of springshed management for sustainable water supply, their crucial role in mountain communities, and the six-step protocol developed by ICIMOD and its partners for reviving the springs with special attention given to the first step – comprehensive mapping of springs and springshed.

### 4.3 GESI inclusive selection of community resource persons (CRPs)

A total of 150 CRPs were selected from 75 wards across seven municipalities and rural municipalities. Two CRPs were chosen from each ward based on criteria such as literacy, smartphone proficiency, survey skills, dedication to mapping springs, and familiarity with the local terrain. The CRP demographic breakdown was 54 % women and 46 % men, with strong representation from indigenous and marginalized communities: Bahun, Chhetri, Thakuri, Sanyasi (BCTS) (39 %); Janajati (39 %); Dalit (11 %); and Newar (11 %). Also, following hardware and software requirements were considered as Survey123 mobile app requires Android 8.0+ or iOS 15+, with at least 2 GB RAM (4 GB recommended) and sufficient storage for offline surveys. A 64-bit ARM processor is preferred for optimal performance. The app needs GPS for location-based data collection and works both online and offline

### 4.4 Training on spring data collection

ArcGIS Survey123 app (version 3.19) with permissions enabled for the camera, location, and other minor features, was used to create a survey questionnaire to collect detailed information of spring sources such as ownership, location, surrounding land use, source condition, water discharge, discharge trends over 10 years, perception of water quality, usage status, household dependency on the spring/pond, cultural/religious significance, the presence of water management user groups, women in leadership positions, and the impact of the 2015 earthquake.

The chosen CRPs underwent a rigorous two-day training program before getting on with field data collection. On the first day, we provided a detailed presentation on springshed management, basic overview of the six-step springshed management protocol, and training on the installation and use of the ArcGIS Survey123 app for mobile-based spring source mapping. Participants acquired practical skills crucial for mapping and data collection using the mobile app.

The second day focused on practical application. We revised training on using the mobile app and addressed all the difficulties, problems and questions raised by the CRPs. This is further enhanced with field visits to the nearby spring site, practicing the use of the app to record detailed spring information, including location and discharge measurements.

### 4.5 Spring source mapping and data verification

After two days of rigorous training, CRPs from the seven municipalities and rural municipalities started to map spring sources. To ensure data accuracy and completeness, a two-step verification process was implemented:

First-step verification: This verification process was carried out in parallel with the field data collection. Researchers from ICIMOD conducted cross-verification of each data point via telephone calls with respective CRPs, which took about 5–6 min per spring. It allowed for the immediate addressing of any issues and correcting mistakes in the collected data and this method was time-efficient and cost-effective.

Second-step verification and validation: This was facilitated by the local government officials. Once the preliminary data was collected and mapped, the maps were sent to each respective ward through the municipality or rural municipality for attribute verification and location and coverage accuracy. Corrections were made as needed, and any missed springs were documented by mobilizing CRPs again. After getting approval from the respective ward, and confirming the accuracy and complete coverage of springs, the process moved to the next step.

This approach facilitated the efficient mapping of a large number of spring resources over an eight-month period, from October 2023 to June 2024.

### 4.6 Documentation of spring sources and spring dashboard

Once spring source data was collected from the seven municipalities and rural municipalities, it was tagged to the HKH Springs Decision Support System (DSS) portal which is an open-access regional database. Regular updating of the dashboard keeps it dynamic and a relevant tool for decision-making and long-term monitoring.

### 4.7 Data used in policymaking and local decision support

In collaboration with all seven municipalities, municipality-wise maps have been created based on the data available in the dashboard. These infographic maps including detailed information on the location and status of spring sources along with social variables were handed over to municipal authorities to assist in planning and prioritizing interventions, particularly in the context of water security.

## Limitations

The Limitations experienced during the data collection process involved inaccessibility and devastating natural disasters. Remote areas with difficult terrain and dense forest make it hard to reach out there for data collection. Due to the landslide and the flood many springs were buried, and the forest fires further disrupted the fieldwork. These limitations may have caused quite minimal gaps in the dataset, thus potentially affecting the accuracy, as well as comprehensiveness, of the findings. To address these challenges in future research, alternative data collection methods or, at least incorporation of resilience measures directly into fieldwork plans to effectively face these difficulties.

## Ethics Statement

Informed consent was verbally obtained from all community participants involved in data collection, who were fully trained for this role.

## CRediT Author Statement

**Srijan Thapa:** Conceptualization, Methodology, Software, Data Curation, Validation, Writing-Original draft. **Anju Pandit:** Conceptualization, Data Curation, Validation, Methodology, Software, Writing - Review & Editing. **Sanjeev Bhuchar:** Conceptualization, Methodology, Writing - Review & Editing, Supervision, Resources, Project administration, Funding acquisition. **Madhav Dhakal:** Conceptualization, Methodology, Writing - Review & Editing, Validation, Supervision, Resources.

## Data Availability

RDS)Status of Springs in Seven Municipalities of Kavre, Nepal (Original data).
